# Posterior mediastinal melanoma causing severe dysphagia: A case report

**DOI:** 10.1186/1752-1947-2-316

**Published:** 2008-09-30

**Authors:** Elisa Meacci, Antonino Mulè, Alfredo Cesario, Claudia Maggiore, Stefano Margaritora

**Affiliations:** 1 Department of Thoracic Surgery, Catholic University, 'Agostino Gemelli' Hospital, Largo A. Gemelli 8 – 00168 Rome, Italy; 2 Department of Pathology, Catholic University, 'Agostino Gemelli' Hospital, Largo A. Gemelli 8 – 00168 Rome, Italy; 3 Pulmonary rehabilitation, IRCCS San Raffaele, Via dellaPisana 235 – 00166 Rome, Italy

## Abstract

**Introduction:**

We describe an original case of progressive severe dysphagia caused by a posterior mediastinal metastatic melanoma of unknown origin. To the best of our knowledge, such an event has never been described before in the literature.

**Case presentation:**

A progressive severe dysphagia case is reported induced by a melanoma of unknown origin (metastatic to a posterior mediastinal lymph node). At the time of diagnosis, the lesion appeared as a large posterior mediastinal mass mimicking a neurogenic tumour with oesophageal involvement. After complete resection, pathological assessment of the tumour by immunohistochemistry was consistent with nodal metastatic melanoma.

**Conclusion:**

This report of a posterior mediastinal lymph node melanoma is unique. The nodal origin is definitely unusual: a primary melanoma should always be carefully ruled out. In fact no other evidence, a part from the absence of the tumour elsewhere, can support the diagnosis of a primary nodal melanoma.

## Introduction

Twenty to thirty percent of all mediastinal tumours are posterior. Most of these (75%) are neurogenic, originating from the neural crest, and often benign (70 to 80%). The remaining histology is rather heterogeneous with lymphoma, teratoma and sarcoma being the most common causes [1].

Symptoms, more often related to malignancies, are usually due to compression or direct invasion of surrounding mediastinal structures and include chest pain, cough, dyspnea or neurological abnormalities. Less frequently, paraneoplastic syndromes can occur.

We describe an original case of progressive severe dysphagia caused by a posterior mediastinal metastatic melanoma of unknown origin.

## Case presentation

A 53-year-old Caucasian man was referred to our centre for absolute dysphagia. This, initiated 4 months before for both solids and liquids, had an insidious onset and was accompanied by a slight cough and persistent fever for which initial antibiotic therapy was prescribed. Fever, usually mild and constantly measured, peaked twice over 40°C.

**Figure 1:**
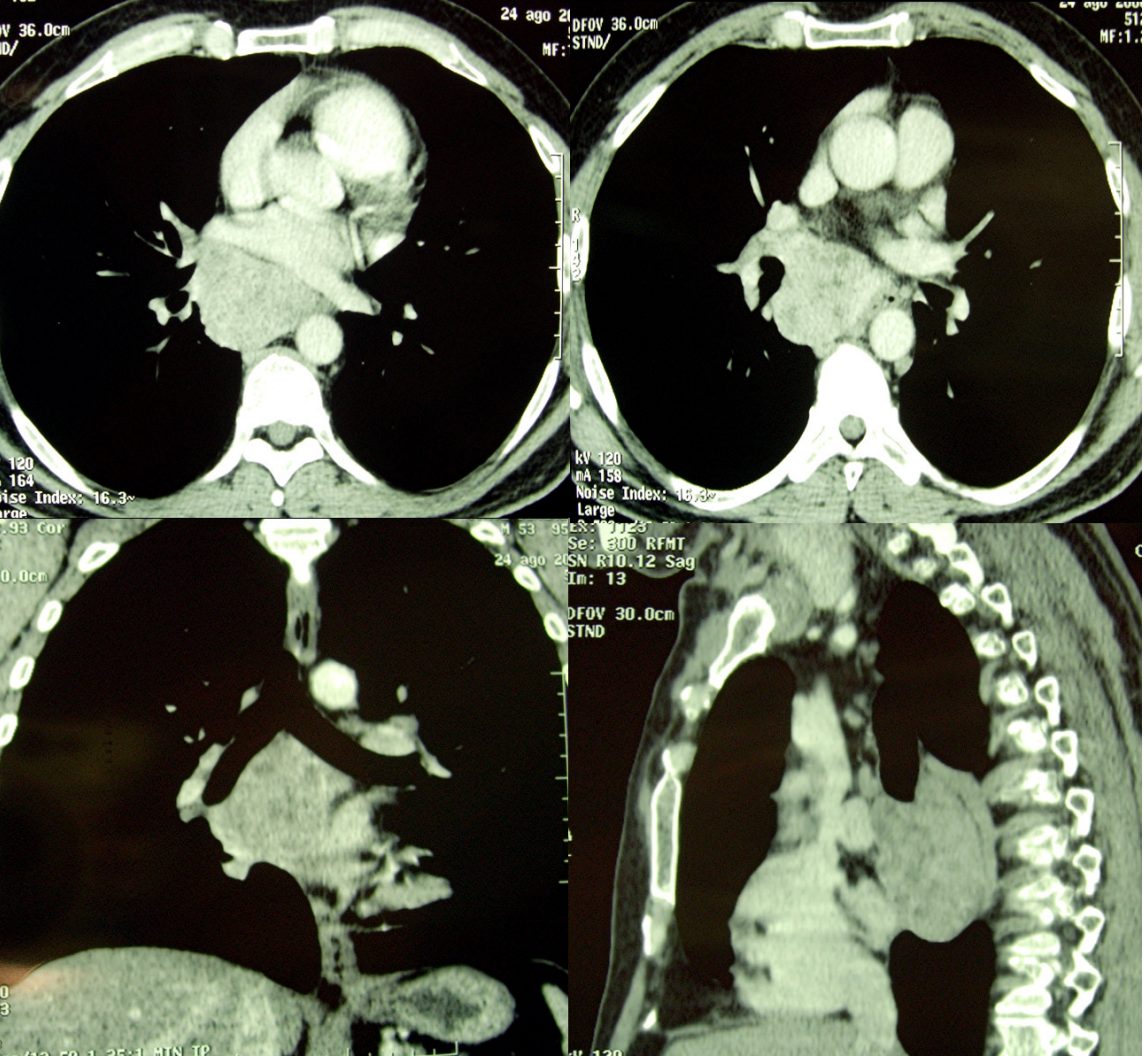
Pre-operative CT scan.

The initial radiological assessment consisted of a chest X-ray showing a large right paratracheal mass. A computed tomography (CT) scan confirmed, at the level of the thorax, the presence of a large (7.5 cm) lobulated mass of heterogeneous density located below the carina. This was clearly compressing the oesophagus. (Figure 1). No evident signs of direct infiltration were found. No other abnormalities were found at the level of the brain, abdomen or pelvis.

A subsequent orogastric endoscopic ultrasonography revealed a hypoechogenic lesion 5 cm in maximum diameter and 25 cm from the incisor teeth, with regular margins, directly compressing the oesophagus. Fine needle aspiration cytology, with double sampling by CT guided transthoracic and ultrasound guided transparietal endoscopic procedures in two different regions of the mass, revealed a loosely dispersed population of rare epithelioid atypical cells with prominent nucleoli and abundant eosinophilic cytoplasm. No lesions or compressions were detected at fibro-tracheo-bronchoscopic examination.

Because of the rapid worsening of symptomatology, the patient underwent surgical intervention with a minimally invasive approach, initially with diagnostic intent. Should resectability have been confirmed, a radical procedure was planned. A right video-assisted thoracoscopic biopsy was performed. The frozen section demonstrated a malignant epithelioid lesion. Lung origin was excluded and further thoracoscopic exploration confirmed the feasibility of a radical resection.

The lesion was radically resected via an open thoracotomy. No signs of direct infiltration of the mass were confirmed at the level of surrounding organs. In particular, the surface of contact with the oesophagus, the right atrium, the main right bronchus and the pulmonary artery was carefully explored. The vagus nerve was identified. A single chest drainage tube was left in situ. The postoperative period was uneventful. The patient started oral intake of fluids on the first postoperative day.

**Figure 2:**
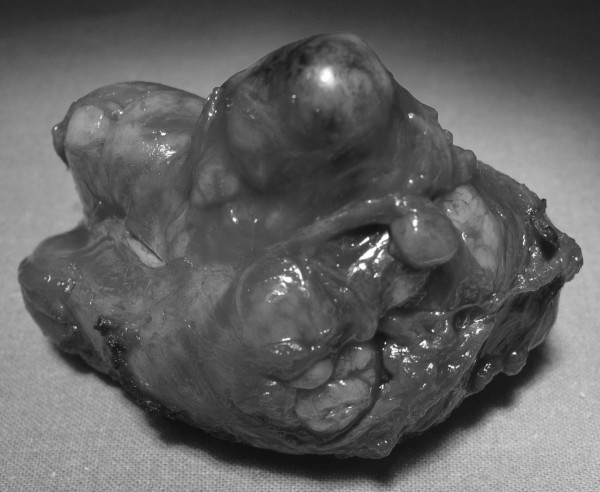
Surgical specimen.

**Figure 3:**
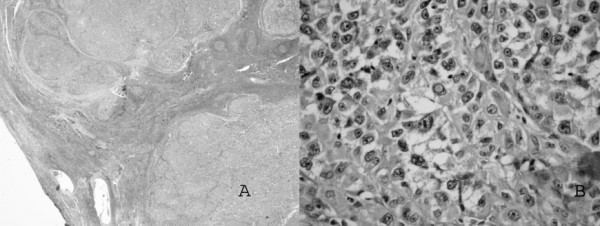
**(A) Solid neoplastic cell sheets separated by a rim of residual lymph node tissue (hematoxylin and eosin, original magnification ×40); (B) Large epithelioid cells with abundant eosinophilic to clear cell cytoplasm. **Prominent nucleoli and focal inclusions are evident in pleomorphic nuclei (hematoxylin and eosin, original magnification ×200).

Gross pathologic examination of the posterior mediastinal mass showed a grey lobulated mass measuring 8 × 9 × 7 cm (Figure 2). Routine histologic studies showed large sheets of epithelioid cells with abundant eosinophilic to clear cytoplasm. Focal spindle cell features and brown pigment were also present. The mass showed peripheral compressed nodal tissue with anthracotic pigment. The nuclei showed frequent inclusions and prominent nucleoli (Figures 3A, B). A Fontana-Masson stain confirmed the presence of melanin pigment in the cytoplasm of neoplastic cells. Immunohistochemical positivity for S-100, Melan A and HMB 45 confirmed the melanomatous nature.

The final diagnosis was malignant metastatic melanoma of a lymph node. No evidence of a primary tumour or superficial nodal involvement was detected outside the mediastinum. The patient is alive and well one year after the operation with no signs of recurrent disease in the mediastinum or appearance of other signs of disease elsewhere. Standard adjuvant immunotherapy has been administered.

## Discussion

Metastatic melanoma arising from an unknown primary site (MUP) was first described in 1952 [2]. It has been estimated that MUP accounts for approximately 1 to 8% of all melanomas [2].

Rare and sparse reports have reported the characteristics of metastatic melanoma occurring as a mediastinal mass. Lau *et al. *[3] discussed the case of a patient with a malignant melanoma presenting as an anterior mediastinal mass consistent with lymph node metastases without evidence of primary melanoma.

To the best of our knowledge, we believe that this case report is unique even considering the large series reported by Baab and McBride [4], where only 4% of the 2446 metastatic melanoma cases described had an unknown site of primary origin. Furthermore, none had the posterior mediastinum as the site of presentation despite the fact that more than half of the patients were admitted with nodal disease only and were treated with regional nodal dissection. Interestingly, the authors supposed that the primary skin lesions had undergone spontaneous regression [4].

Metastasis to the mediastinal lymph node generally originates from primary thoracic malignancies. In the natural history of extrathoracic malignancies, pulmonary parenchymal metastases are more common than metastatic involvement of mediastinal lymph nodes [5]. Melanoma nodal metastasis is not an event occurring randomly. In fact, often an orderly and sequential manner is respected where the involvement of the sentinel node(s) occurs first, and the involvement of higher level nodes later. Skip metastases are very rare [6].

In our case, the absence of a primary malignancy together with the lack of skin nodal basin involvement suggested a nodal origin rather than a metastatic localization from a skin or mucosal lesion which underwent spontaneous regression The focal presence of residual nodal tissue excluded the exceptional possibility of a clear cell sarcoma arising from mediastinal soft tissue.

For the sake of completeness, it is worth mentioning that benign nevus cell aggregates in lymph nodes are believed to be able to instigate primary nodal malignant melanoma, without an obvious extranodal site of origin. [7]

## Conclusion

This report of a posterior mediastinal lymph node melanoma is unique. The nodal origin is definitely unusual: a primary melanoma should always be carefully ruled out. In fact no other evidence, a part from the absence of the tumour elsewhere, can support the diagnosis of a primary nodal melanoma.

## Competing interests

The authors declare that they have no competing interests.

## Authors' contributions

EM was the principal investigator in the study, operated on the patient, and was involved in drafting the manuscript. AM performed the interpretation of stereomicroscopic information, compiled the technical report and was involved in drafting the article; CM performed the interpretation of stereomicroscopic information, compiled the technical report and was involved in drafting the article; SM operated upon the patient, helped in manuscript drafting and in the collection of the literature. AC has been involved in drafting the manuscript (reviewed the literature and completed the drafting of the manuscript) and gave final approval of the version to be published. All of the authors read and approved the final manuscript.

## Consent

Written informed consent was obtained from the patient for publication of this case report and any accompanying images. A copy of the written consent is available for review by the Editor-in-Chief of this journal.
